# The effect of *Saccharomyces boulardii* supplementation on *Helicobacter pylori* eradication in children: a systematic review and meta-analysis of Randomized controlled trials

**DOI:** 10.1186/s12879-023-08896-4

**Published:** 2023-12-15

**Authors:** Lian-Hua Liu, Bin Han, Jing Tao, Kai Zhang, Xi-Ke Wang, Wen-Yu Wang

**Affiliations:** 1 Department of Pediatric Gastroenterology, Guizhou Branch of Shanghai Children’s Medical Center, Shanghai Jiaotong University School of Medicine, Guizhou provincial people’s hospital, Guiyang, Guizhou Province China; 2 Department of Endoscopy, Guizhou Branch of Shanghai Children’s Medical Center, Shanghai Jiaotong University School of Medicine, Guizhou provincial people’s hospital, Guiyang, Guizhou Province China; 3 Department of Pediatric Gastroenterology, Guizhou Branch of Shanghai Children’s Medical Center, Shanghai Jiaotong University School of Medicine, 395 Jinzhu East Road, Guanshanhu District, Guiyang City, Guizhou Province China

**Keywords:** *Helicobacter pylori*, *Saccharomyces boulardii*, Probiotics, Meta-analysis, Systematic review, Randomized controlled trials

## Abstract

**Background:**

It is unclear whether *Saccharomyces boulardii* (*S. boulardii*) supplementation in standard triple therapy (STT) is effective in eradicating *Helicobacter pylori* (*H. pylori*) infection in children. We therefore conducted a meta-analysis of randomized controlled trials (RCTs) to assess the effect of *S. boulardii* supplementation on *H. pylori* eradication in children.

**Methods:**

We conducted electronic searches in PubMed, Embase, the Cochrane Library, China National Knowledge Infrastructure and Wanfang database from the beginning up to September 2023. A random-effects model was employed to calculate the pooled relative risk (RR) with 95% confidence intervals (CI) through a meta-analysis.

**Results:**

Fifteen RCTs (involving 2156 patients) were included in our meta-analysis. Results of the meta-analysis indicated that *S. boulardii* in combination with STT was more effective than STT alone (intention-to-treat analysis : 87.7% vs. 75.9%, RR = 1.14, 95% CI: 1.10–1.19, *P* < 0.00001; per-protocol analysis : 88.5% vs. 76.3%, RR = 1.15, 95% CI: 1.10–1.19, *P* < 0.00001). The *S. boulardii* supplementation group had a significantly lower incidence of total adverse events (n = 6 RCTs, 9.2% vs. 29.2%, RR = 0.32, 95% CI: 0.21–0.48, *P* < 0.00001), diarrhea (n = 13 RCTs, 14.7% vs. 32.4%, RR = 0.46, 95% CI: 0.37–0.56, *P* < 0.00001), and nausea (n = 11 RCTs, 12.7% vs. 21.3%, RR = 0.53, 95% CI: 0.40–0.72, *P* < 0.0001) than STT group alone. Similar results were also observed in the incidence of vomiting, constipation, abdominal pain, abdominal distention, epigastric discomfort, poor appetite and stomatitis.

**Conclusions:**

Current evidence indicated that *S. boulardii* supplementing with STT could improve the eradication rate of *H. pylori*, and concurrently decrease the incidence of total adverse events and gastrointestinal adverse events in children.

**Supplementary Information:**

The online version contains supplementary material available at 10.1186/s12879-023-08896-4.

## Introduction

*Helicobacter pylori* (*H. pylori*), a microaerobic gram-negative bacterium that colonizes the gastric mucosa, is a very common infection across the world and is a significant public health problem [1]. According to the latest meta-analysis study, the rate of *H. pylori* infection in developing countries was found to be 50.8%, 34.7% in developed countries, and 32.6% for children worldwide [2]. *H. pylori* is usually contracted in early childhood and, if not addressed, can remain for a long time [3]. It is generally believed that *H.pylori* infection is the primary cause of chronic gastritis, peptic ulcers, and gastric malignancies [3, 4]. Additionally, recent studies have indicated a possible correlation between *H.pylori* infection and some extra-gastroduodenal diseases (cardiovascular diseases, metabolic disorders, diabetes mellitus, neurodegenerative diseases, and skin diseases) [4]. Eliminating *H.pylori* is the most effective way to prevent and treat illnesses associated with *H.pylori*.

The standard triple therapy (STT) based on a proton pump inhibitor (PPI) combined with two antibiotics (amoxicillin and clarithromycin or metronidazole) was the first-line regimen for *H. pylori* eradication in children in several recent guidelines and expert consensus for eradicating *H. pylori* [5–7]. However, due to the increasing resistance rate of antibiotics (mainly clarithromycin), the eradication rate has decreased. Additionally, the adverse events of antibiotics during the eradication process can lead to poor compliance [1, 8–10]. Therefore, new effective therapies are needed to eradicate *H. pylori*. Scientists have started to explore other alternative and complementary treatments to tackle these issues.

Over the past few years, probiotics have been extensively utilized in clinical practice, providing a novel approach for eradicating *H. pylori* [8–10]. Probiotics, live microorganisms that can be advantageous to one’s wellbeing when taken in the right amount, are a type of beneficial microorganism [11]. *Saccharomyces boulardii* (*S. boulardii*) is the only fungal probiotic preparation that is being employed around the world. Previously, Zhou et al. [12] conducted a meta-analysis to explore the effect of *S. boulardii* in combination with standard eradication therapy (triple therapy, quadruple therapy and sequential therapy) on *H. pylori* eradication. Nevertheless, the previous meta-analysis mainly concentrated on adults, with only three studies on children having small sample sizes. What is more, recent several guidelines and expert consensus on *H. pylori* eradication in children have highlighted the absence of adequate clinical evidence to back up the regular inclusion of probiotics during *H. pylori* eradication therapy [5–7]. There has yet to be a definitive conclusion concerning the effectiveness of using *S. boulardii* to treat *H. pylori* infection. Hence, we conducted a systematic review and meta-analysis of randomized controlled trials (RCTs) to evaluate the effect of *S. boulardii* supplementing with STT on *H. pylori* eradication in children and provide evidence-based medical evidence for clinical decision-making.

## Methods

This study has been pre-registered in PROSPERO, which is an International Prospective Register of Systematic Reviews and Meta-Analyses (registration number. CRD42023462632). This systematic review followed the PRISMA 2020 statement for Systematic Reviews and Meta-Analysis [13].

### Literature search strategy

We conducted electronic searches in PubMed, Embase, the Cochrane Library, China National Knowledge Infrastructure (CNKI) and Wanfang database using pre-determined search terms from the beginning up to September 10, 2023.The terms used for the search were: *helicobacter*, “*helicobacter pylori*”, “*campylobacter pylori*”, “*H.pylori*”, “Hp”, “*saccharomyces boulardii*”, “*S. boulardii*”, “*bioflor*”, probiotics, probiotic, “children”, “childhood”, “pediatric”, “pediatrics”, “adolescents”. For details on search terms and strategies used in PubMed, please refer to the online Supplementary Table S1. We employed MeSH headings and text word terms searching without any language limitations. Furthermore, we scanned the reference lists of evaluated studies to find additional suitable studies.

### Study selection criteria

The following criteria were used to determine which studies were eligible: (1) Study design: RCTs; (2) Participants: Participants in the study were first-time treatment patients with *H.pylori* infection (aged < 18 years old), which was determined through urea breath test (UBT), rapid urease test (RUT), histology, stool antigen test (SAT), or culture, with one positive result at least; (3) Intervention group (*S. boulardii* supplementation group): *S. boulardii* in combination with STT; (4) Control group (STT group): the same STT as intervention group (with or without placebo); (5) Outcome: (i) primary outcome: *H.pylori* eradication rate (after ceasing medication for at least 4 weeks, re-examination using established *H.pylori* testing methods, and a negative result for *H.pylori* eradication); (ii) secondary outcomes: the incidence of adverse events (including the total and specific adverse events). Studies involving other probiotics, adults, repeated published research, non-RCTs, conference abstracts, letters, animal experiments, meta-analyses, reviews, and studies where data or full text cannot be obtained will be excluded. After reviewing titles, abstracts and full texts of the obtained literatures, two researchers (Liu LH and Tao J) independently excluded any studies that did not meet the inclusion criteria. In case of any disagreement, consensus was reached through discussion or with the help of a third researcher.

### Data extraction

Two researchers (Zhang K and Wang WK) were delegated to independently extract data, using a unified data extraction form. Agreement on data extraction was established by discussion.The data extracted from the eligible studies was as follows: the first author, publication year, country, sample size, age, diagnostic methods of *H. pylori* (initial/rechecking), details of the intervention group and the control group, and details of outcomes of interest.

### Risk of bias and grading the strength of evidence

Two researchers (Liu LH and Tao J) separately evaluated the risk of bias and quality of evidence for each study, and consensus was used to settle any disputes. The Cochrane Collaboration’s tool was employed to assess the risk of bias of the included studies. The main domains of evaluation were random sequence generation, allocation concealment, blinding of participants and personnel, blinding of outcome assessment, incomplete outcome data, selective reporting, and other biases [14]. We evaluated each domain based on three categories (low risk, high risk, and uncertain risk). Utilizing the Grading of Recommendations, Assessment, Development and Evaluation (GRADE) approach and GRADE profiler software (Version 3.6.1, McMaster University, 2014), the quality of evidence was appraised and categorized into four levels: high, moderate, low, and very low [15].

### Statistical analysis

Meta-analysis was performed utilizing RevMan5.3 software (Cochrane Collaboration, Copenhagen, Denmark), and relative risk (RR) and its 95% confidence intervals (CIs) were employed as efficacy analysis statistics for counting data.

To get a conservative estimate of the 95% CI, we utilized a random-effects model to analyze the data for all outcomes [16]. For *H.pylori* eradication rate, data were analyzed by intention-to-treat (ITT) and per-protocol (PP) analysis. Assessing the heterogeneity between studies, the I^2^ statistic and the chi-square test with a *P* value < 0.10 were used, the latter to define substantial heterogeneity. Heterogeneity was classified as insignificant, low, moderate, and high when I^2^ values were 0–25%, 26–50%, 51–75%, and above 75%, respectively [17]. For primary outcome (*H.pylori* eradication rate), subgroup analyses was conducted based ITT analysis. To determine whether the exclusion of any single study would significantly alter the results of the remainders, sensitivity analyses were conducted by omitting each trial one at a time. An evaluation of publication bias was conducted with the aid of funnel plot, Begg’s test [18] and Egger’s test [19], which were all implemented using STATA/SE (Version 12.0, STATA Corporation, Texas, USA).

## Results

### Study selection

**Fig. 1:**
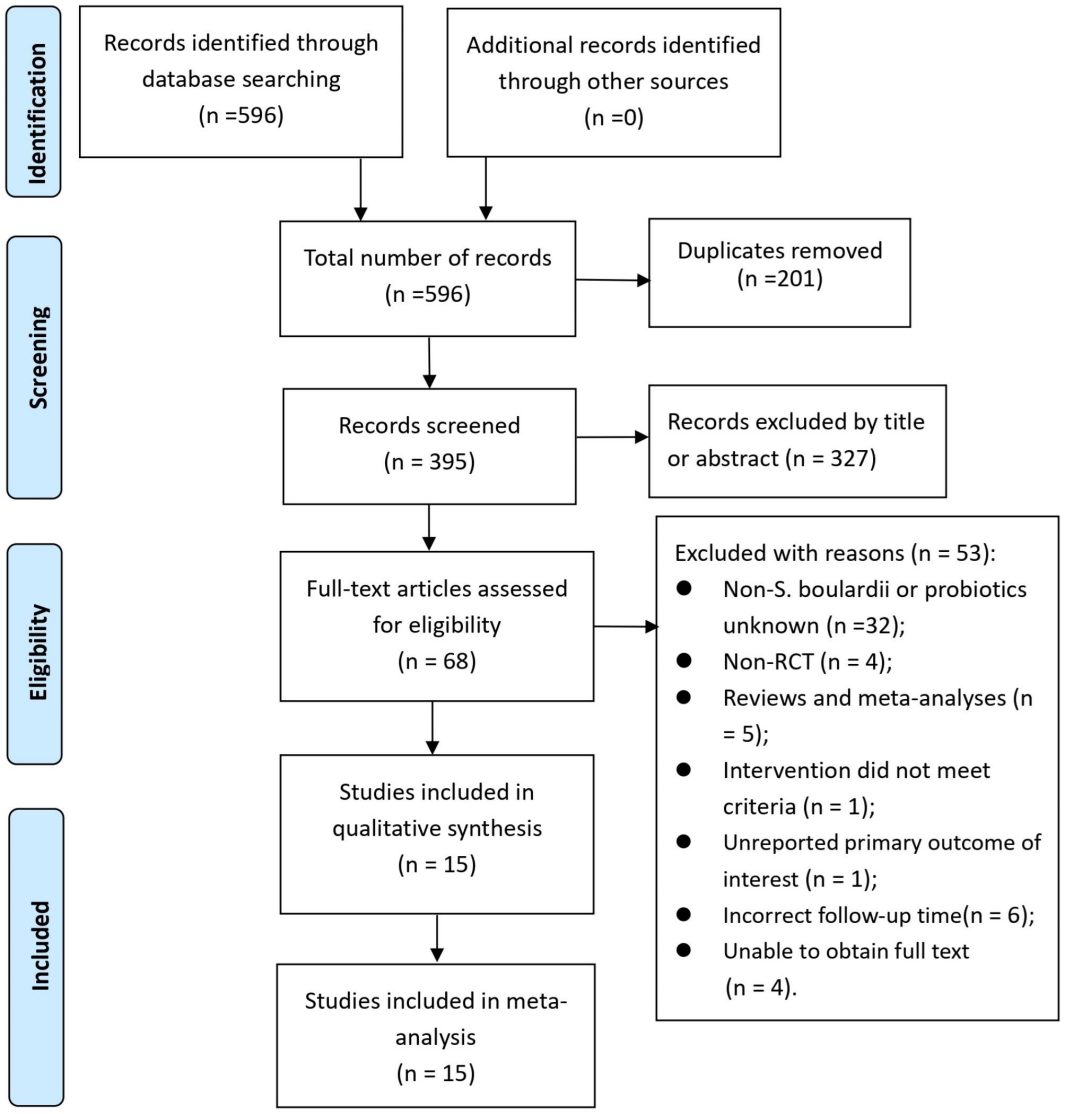
PRISMA flowchart of study selection process

The literature search produced 596 records. After eliminating 201 duplicate articles, 327 were further excluded from the 395 articles by reading titles or abstracts. Subsequently, 68 articles were assessed in full text, out of which 53 were excluded for various reasons (see Supplementary Table S2). Consequently, 15 studies [20–34] were included in this meta-analysis. A visual representation of the multi-step selection process was depicted in Fig. 1.

### Study characteristics

The 15 RCTs (involving 2156 patients) included were conducted between 2009 and 2023, with one [20] in Europe (Romania) and the rest in Asia (China) [21–34]. The sample size of the trials spanned from 60 to 500 individuals. Regarding *H. pylori* eradication regimens, the vast majority of the regimens were standard triple therapy (omeprazole + amoxicillin + clarithromycin), and the duration of eradication regimen vary from a week to two weeks. Most studies have included *S. boulardii* with a dosage of 500 mg/day for a period of 14 days. Table 1 outlines the basic characteristics of the studies that were included in the meta-analysis.

**Table 1:** Characteristics of included studies in the meta-analysis

Study	Country	No. of patients (Exp/Con)	Age (years)	Eradication regimen	*S.boulardii* regimen	Diagnositic methods of *H. pylori*	
						Initial	Rechecking
Hurduc 2009	Romania	90 (48/42)	3~18	Omeprozole / esomeprazole + clarothromycin + amoxicillin, 7–10 days	250 mg bid, 28 days	RUT, histology	RUT, histology
Zhang 2012	China	82 (41/41)	11.13 ± 2.69/10.04 ± 2.10	Omeprazole + amoxicillin + clarithromycin, 14 days	250 mg qd, 7 days	Histology, RUT, UBT	UBT
Zhang 2013	China	60 (30/30)	9.82 ± 3.27/10.15 ± 3.34	Omeprazole + amoxicillin + clarithromycin, 14 days	250 mg bid, 14 days	UBT	UBT
Zhao 2014	China	240 (120/120)	7 ± 2/9 ± 2	Omeprazole + amoxicillin + clarithromycin, 14 days	250 mg bid, 14 days	RUT + UBT	UBT
Zhou 2015	China	116 (58/58)	8.91 ± 3.24/ 9.02 ± 3.16	Omeprazole + amoxicillin + clarithromycin, 10 days	125 mg bid, 10 days	RUT + UBT	UBT
Bin 2015	China	205 (105/100)^a^	13.33 ± 1.26	Omeprazole + clarithromycin + amoxicillin / metronidazole, 14 days	500 mg/day, 14 days	Histology, serology	UBT
Chen 2015	China	240 (120/120)	8.9 ± 1.9/9.6 ± 2.1	Omeprazole + amoxicillin + clarithromycin, 14 days	250 mg bid, 14 days	RUT + UBT	RUT + UBT
Wang 2017	China	107 (57/50)	9 ± 5	Omeprazole + amoxicillin + clarithromycin, 14 days	250 mg qd, 14 days	UBT	UBT
Xiang 2017	China	500 (250/250)	7.25 ± 2.16	Omeprazole + amoxicillin + clarithromycin, 14 days	250 mg bid, 14 days	UBT + RUT	UBT
Dong 2018	China	160 (80/80)	5~14	Omeprazole + amoxicillin + clarithromycin, 14 days	250 mg bid, 14 days	UBT + histology	UBT
He 2019	China	120 (60/60)	7.8 ± 2.1/ 9.2 ± 2.4	Omeprazole + amoxicillin + clarithromycin, 14 days	250 mg bid, 14 days	SAT or UBT	UBT
Zhu 2019	China	80 (40/40)	3~10	Omeprazole + amoxicillin + clarithromycin, 14 days	250 mg bid, 14 days	SAT or UBT	SAT or UBT
Xiao 2021	China	90 (45/45)	9.77 ± 2.32/10.23 ± 2.51	Omeprazole + amoxicillin and clavulanate potassium + clarithromycin, 14 days	250 mg bid, 14 days	RUT	UBT
Zhang 2021	China	150 (100/50)	3~14	Omeprazole + amoxicillin + clarithromycin, 14 days	250 mg bid, 14 days	Histology, or culture or RUT	UBT
Liu 2023	China	79 (40/39)	10.18 ± 2.12/10.36 ± 1.98	Omeprazole + amoxicillin + clarithromycin, 14 days	250 mg bid, 14 days	Histology, culture + RUT	UBT

Exp, experimental group (*S. boulardii* + standard triple therapy); Con, control group (standard triple therapy); qd, once daily; bid, twice daily; UBT, Urea breath test;RUT, rapid urease test; SAT, stool antigen test; ^a^ 42 children were tested four weeks after the end of the eradication therapy to confirm eradication.

### Risk of bias

Of the 15 RCTs, 7 RCTs [20, 24, 28, 30, 31, 33, 34] were assessed as low risk of randomization due to their sufficient random sequence generation methods, while the remaining 8 RCTs [21–23, 25–27, 29, 32] were deemed unclear risk. Regarding blinding, one study [20] was an open trial and was judged to be of high risk, the other 14 RCTs were considered to have an unclear risk of bias due to a lack of adequate information. All studies failed to provide information for allocation concealment, making the risk of bias unclear. All studies indicated low risk in terms of incomplete outcome data, selective reporting, and other bias. A thorough examination of the risk of bias can be found in Supplementary Figures S1 and S2.

### Primary outcome: *H. Pylori* eradication rate

Fifteen RCTs provided information on the *H. pylori* eradication rate. Results of the meta-analysis indicated that

**Fig. 2:**
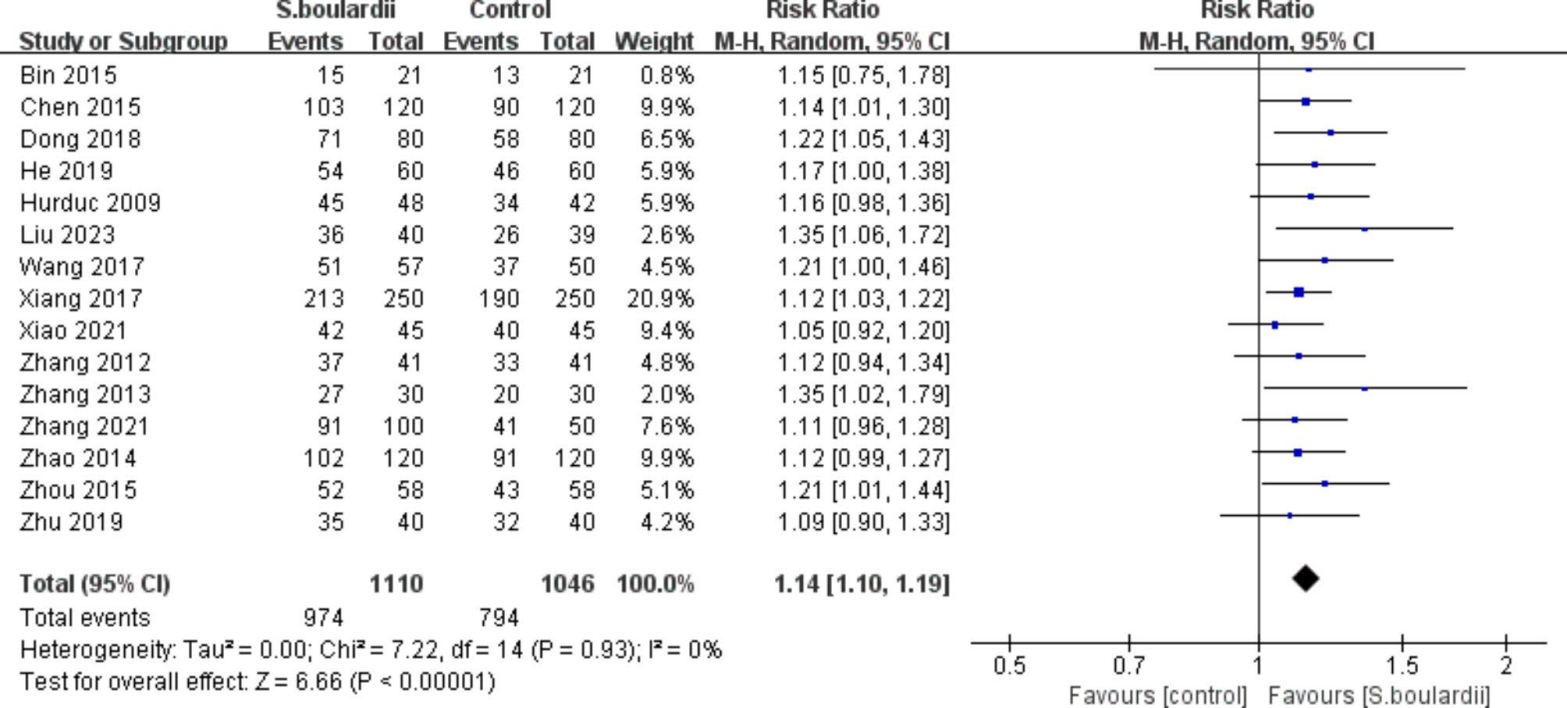
Forest plot for the effect of *S. boulardii* supplementation on *H. pylori* eradication rate (ITT data)

**Fig. 3:**
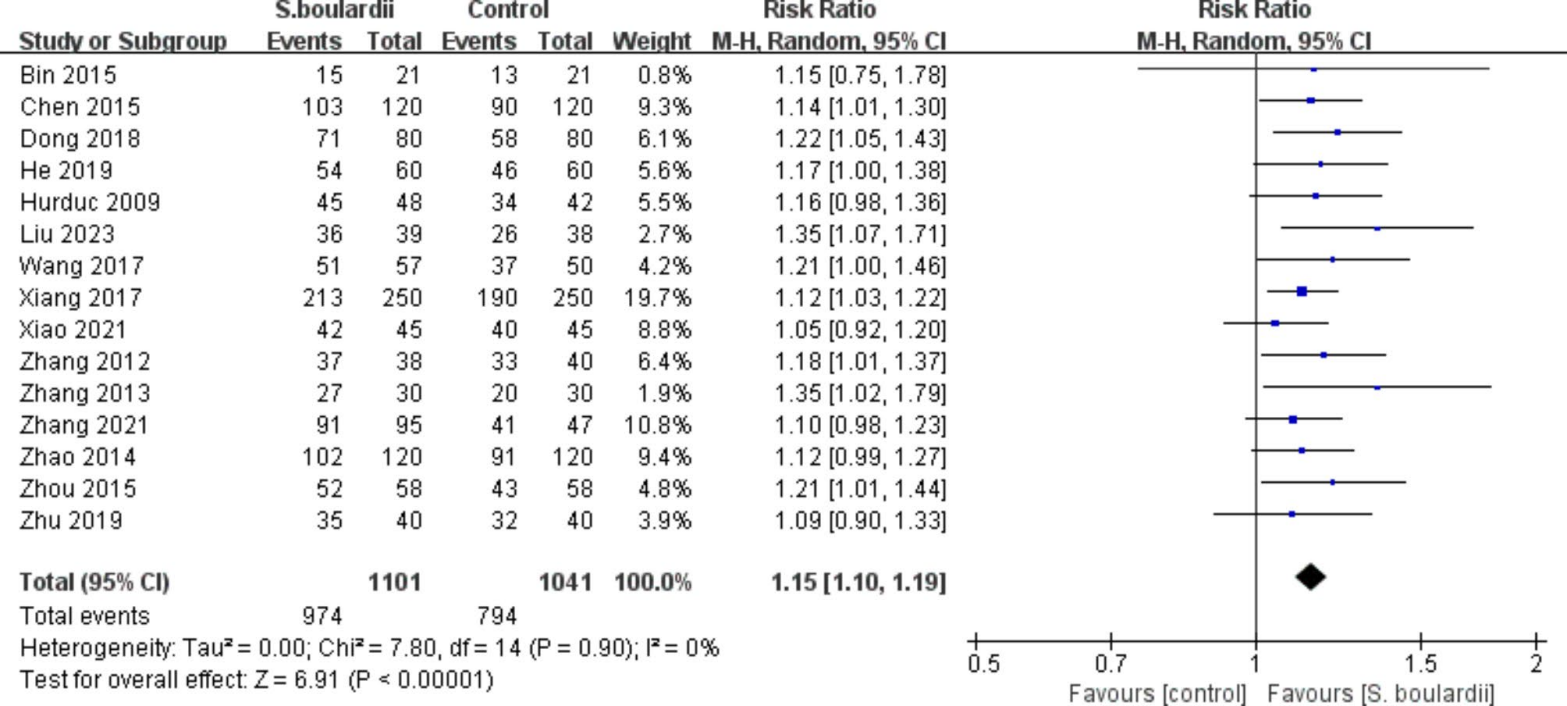
Forest plot for the effect of *S. boulardii* supplementation on *H. pylori* eradication rate (PP data)

*S. boulardii* in combination with STT was more effective than STT alone, regardless of ITT analysis (87.7% vs. 75.9%, RR = 1.14, 95% CI: 1.10–1.19, *P* < 0.00001) (Fig. 2) or PP analysis (88.5% vs. 76.3%, RR = 1.15, 95% CI: 1.10–1.19, *P* < 0.00001) (Fig. 3). No heterogeneity was identified across studies (I^2^ = 0).

**Fig. 4:**
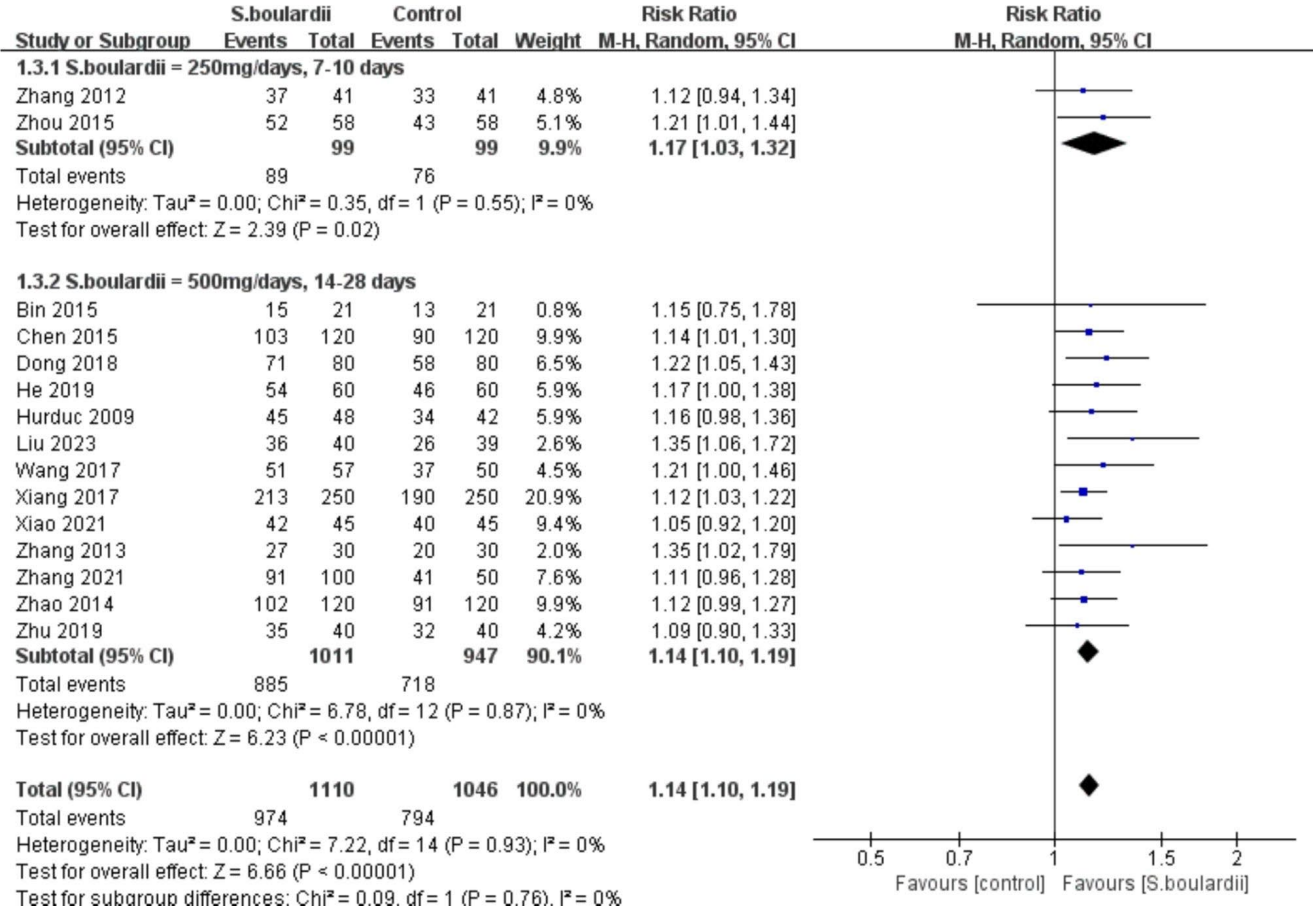
Forest plot for subgroup analysis based on the dose and duration of *S. boulardii* on *H. pylori* eradication rate

Subgroup analyses based on the dose and duration of *S. boulardii* were conducted to explore the potential influencing factor on the overall results. The results indicated that *S. boulardii* supplementation significantly improved *H. pylori* eradication rate in both low dose and duration *S. boulardii* group (250 mg/days, 7–10 days) (n = 2 RCTs, RR = 1.17, 95%CI:1.03-1.32, *P* = 0.02) and high dose and duration *S. boulardii* group (500 mg/days, 14–28 days) (n = 13 RCTs, RR = 1.14, 95%CI:1.10–1.19, *P* < 0.00001) (Fig. 4). Performing a sensitivity analysis by omitting one trial at a time revealed that the pooled results of the remaining studies remained unchanged, indicating the results were stable.

### Secondary outcomes: adverse events

**Fig. 5:**
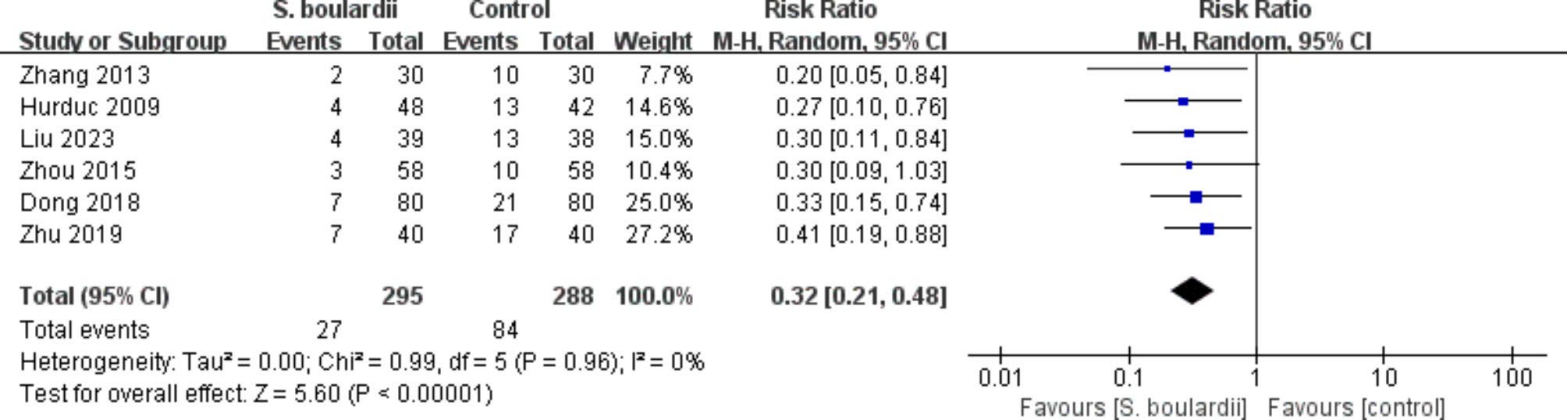
Forest plot for the effect of *S. boulardii* supplementation on total adverse events

Data on total adverse events was reported in six RCTs [20, 22, 24, 29, 31, 34] involving 583 patients. The meta-analysis revealed that the combination of *S. boulardii* and STT had a significantly lower incidence of total adverse events than STT alone (9.2% vs. 29.2%, RR = 0.32, 95% CI: 0.21–0.48, *P* < 0.00001). No heterogeneity was observed (I^2^ = 0, *P* = 0.96) (Fig. 5).

**Fig. 6:**
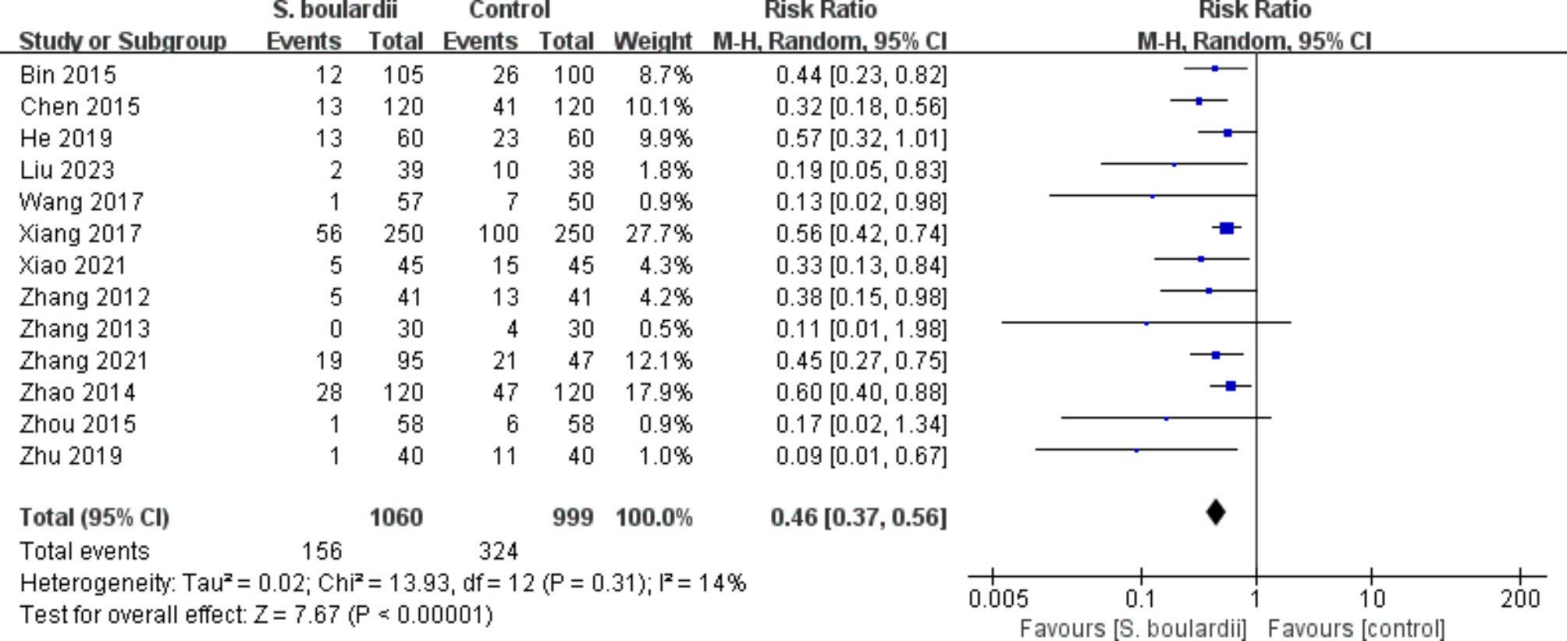
Forest plot for the effect of *S. boulardii* supplementation on diarrhea

**Fig. 7:**
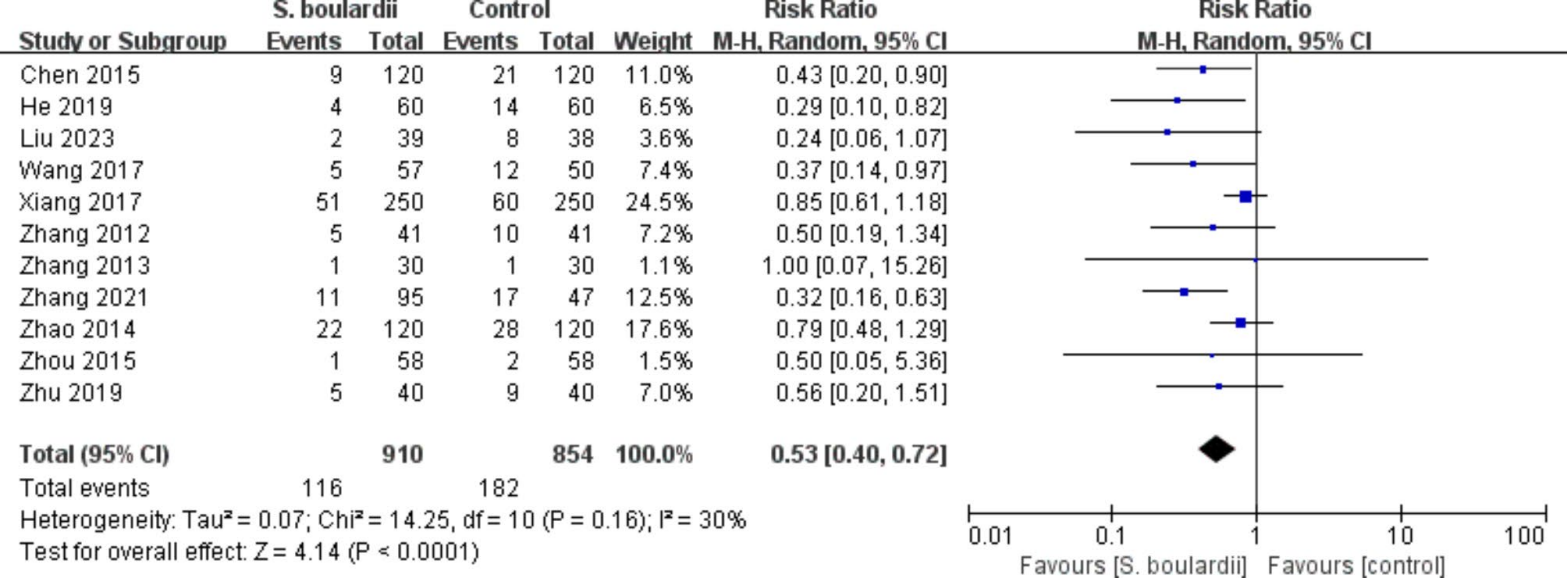
Forest plot for the effect of *S. boulardii* supplementation on nausea

Regarding specific adverse events, the meta-analysis revealed that *S. boulardii* combined STT group had a significantly lower incidence of diarrhea (n = 13 RCTs, 14.7% vs. 32.4%, RR = 0.46, 95% CI: 0.37–0.56, *P* < 0.00001) (Fig. 6) and nausea (n = 11 RCTs, 12.7% vs. 21.3%, RR = 0.53, 95% CI: 0.40–0.72, *P* < 0.0001) (Fig. 7) when compared to the STT group. Similarly, the incidence of vomiting, constipation, abdominal pain, abdominal distention, epigastric discomfort, poor appetite and stomatitis in the *S. boulardii* combined with STT group was significantly lower than that in the STT group (Supplementary Figure S3-S4). However, we did not observe any considerable difference between the research groups in terms of taste disorder (n = 1 RCT, RR = 0.50, 95% CI: 0.16–1.53, *P* = 0.22) and rash (n = 4 RCTs, RR = 0.32, 95% CI: 0.09–1.18, *P* = 0.09). The results of adverse events can be seen in Table 2.

**Table 2:** Effect of S. Boulardii plus standard triple therapy vs. standard triple therapy on adverse events

Adverse events	Number of studies	Sample size	RR (95%CI)	*P*_effect_	*I*^*2*^(%)	*P*_heterogeneity_
Total adverse events	6	583	0.32 (0.21–0.48)	<0.00001	0	0.96
Diarrhea	13	2 059	0.46 (0.37–0.56)	<0.00001	14	0.31
Nausea	11	1 764	0.53 (0.40–0.72)	<0.0001	30	0.16
Vomiting	6	1 257	0.67 (0.47–0.94)	0.02	58	0.04
Constipation	6	1 099	0.42 (0.31–0.58)	<0.00001	0	0.83
Abdominal pain	6	1 319	0.67 (0.46–0.96)	0.03	43	0.12
Abdominal distention	4	408	0.47 (0.27–0.81)	0.006	0	0.53
Epigastric discomfort	2	198	0.41 (0.18–0.90)	0.03	0	0.50
Poor appetite	6	706	0.50 (0.35–0.72)	0.0002	13	0.33
Taste disorder	1	82	0.50 (0.16–1.53)	0.22	-	-
Stomatitis	2	740	0.26 (0.15–0.47)	<0.00001	0	0.80
Rash	4	382	0.32 (0.09–1.18)	0.09	0	0.97

RR, relative risk; CI, confidence interval.

### GRADE assessment

We employed the GRADE approach to assess the quality of evidence. According to the GRADE assessment, the *H. pylori* eradication rate (ITT data) has a low quality rating, largely due to the potential for bias stemming from unclear random sequence generation and allocation concealment, no or unclear blinding, and reporting bias.The quality of evidence for the incidence of all adverse events was assessed as low to medium, mainly because of risk of bias in study design, the possibility of bias, statistical heterogeneity, or reporting bias. The GRADE evidence profiles for all the outcomes can be found in Supplementary Tables S3-S4.

### Publication bias

An examination of publication bias was conducted on three outcomes (*H. pylori* eradication rate, incidence of diarrhea and nausea) that had more than 10 studies, and the visual funnel plot revealed a slight asymmetry (Supplementary Figure S5-S7). Furthermore, we utilized Begg’s test and Egger’s test for quantitative detection of publication bias. For *H. pylori* eradication rate, Begg’s test did not detect any evidence of publication bias (*P*_Begg_ = 0.06), but Egger’s test suggested that there may be a publication bias (*P*_Egger_ = 0.034). Similar results were observed for nausea (*P*_Begg_ = 1.000, *P*_Egger_ = 0.041). For diarrhea, results from both Begg’s test and Egger’s test indicate the possibility of publication bias (*P*_Begg_ = 0.009, *P*_Egger_ = 0.000).

## Discussion

Currently, probiotics are commonly supported for their positive effects on human health and are frequently employed to treat antibiotic-induced diarrhea, inflammatory bowel disease, irritable bowel syndrome, allergic illnesses, and cancer [1, 35]. With the deepening of research into intestinal microecology in recent years, the concept of “probiotics inhibiting bacteria” has brought forth new ideas for eradicating *H. pylori*. *S. boulardii* is a non-pathogenic yeast that is able to resist stomach acid, bile, and proteolytic enzymes. Compared to other bacterial probiotics, its major distinction is its inherent resistance to antibiotics, making it suitable for use in conjunction with antibiotics [36, 37]. It is unclear exactly how

*S. boulardii* eliminates *H. pylori*, but there are a few possible explanations. One is that it produces substances, such as short chain fatty acids, that inhibit *H. pylori* growth [38]. Additionally, *S. boulardii* has a larger surface area than other probiotics, so it can better adhere to the gastric mucosa, preventing *H. pylori* colonization and adhesion [37]. The neuraminidase present in *S. boulardii* can also remove *H. pylori* adhesin ligands and stop the bacteria from attaching to the duodenal mucosa [39]. Furthermore, *S. boulardii* has been shown to stabilize the tight junction of gastric mucosal epithelial cells, stimulating SIgA response and strengthening the gastric mucosal barrier [40, 41]. Finally, by reducing adverse reactions and improving patient compliance, *S. boulardii* may indirectly improve the eradication rate of *H. pylori*.

As far as we are aware, this is the first meta-analysis to explore the effect of *S. boulardii* supplementing with STT in eradicating *H. pylori* in children. In this meta-analysis of 15 RCTs (2156 children), we found that *S. boulardii* was beneficial in improving the *H. pylori* eradication rate of STT, which was consistent with the findings of the previous meta-analysis [12]. However, the prior meta-analysis took into account 18 RCTs, mostly concerning adults, with only three of the RCTs involving children. Our meta-analysis focused solely on children, and all the RCTs included in previous studies were included in our meta-analysis. Thus, the conclusions drawn are more comprehensive and reliable. Antibiotics may disrupt the balance of the gastrointestinal microbiota, resulting in gastrointestinal adverse events such as diarrhea, nausea, vomiting, abdominal pain, and abdominal distention. This study found that the addition of *S. boulardii* reduced the total incidence of adverse events by 20% and decreased the incidence of major gastrointestinal adverse events such as diarrhea, nausea, vomiting, constipation, abdominal pain, abdominal distention, epigastric discomfort, and poor appetite.

Nowadays, *H. pylori* infection has become a major public health concern. Treating *H. pylori* is becoming more difficult due to the rising antibiotic resistance and patient noncompliance [42]. Our research has significant clinical implications as it provides a good treatment strategy for *H. pylori* infection, i.e. supplementing with *S. boulardii* in STT can significantly improve the eradication rate of *H. pylori* and reduce some adverse events during eradication treatment. Despite the lack of strong evidence (low evidence for *H. pylori* infection rate and low to medium evidence for adverse events) to support its widespread recommendation, physicians should be cognizant of these strategies when attempting to eradicate *H. pylori*.

Our meta-analysis has several strengths. Our meta-analysis collected data limited to a single probiotic, *S. boulardii*, as it appears not all probiotics are effective in eradicating *H. pylori*. To our knowledge, this is the first meta-analysis to explore the efficacy of supplementing *S. boulardii* with STT in eradicating *H. pylori* only in the pediatric population. In addition, we employed a thorough search strategy, explicit selection criteria, and a stringent quality assessment with the aid of the Cochrane Collaboration tool and GRADE approach, in addition to strictly adhering to the PRIMSA statement for reporting, with no heterogeneity detected among estimates for primary outcome. Hence, our meta-analysis provided the most comprehensive and convincing evidence for the efficacy of the STT for the elimination of *H. pylori* in children with *S. boulardii*.

Despite its strengths, this meta-analysis has certain limitations that should be taken into consideration. Firstly, this meta-analysis only included RCTs, however, most studies had obscured allocation and uncertain blinding, which could have an effect on subjective outcome indicators, such as the incidence of adverse events. In the future, it is necessary to conduct open and transparent RCTs with large samples and high-quality multicenter placebo controls for further exploration of the effect of *S. boulardii* in assisting in eradicating *H. pylori.* Secondly, most of the studies were conducted in Asia (China), while one was conducted in Europe (Romania). Given the antibiotic resistance patterns of *H. pylori* infection and the potential variability of population characteristics, additional research is necessary in Western countries in the future. Thirdly, there were certain differences in the eradication regimen and treatment durations and doses of *S. boulardii* among different studies, but this was only a small part of the included studies. The majority of the eradication regimens in most studies were omeprazole + amoxicillin + clarithromycin, and most studies studies have included *S. boulardii* with a dosage of 500 mg/day for a period of 14 days. Therefore, we believe that the impact on our results can be negligible. Finally, our publication bias test indicated the possibility of publication bias. We have adopted a wide range of retrieval strategies, including papers and conference abstracts, to reduce the possibility of such bias.

## Conclusion

To conclude, current evidence indicated that *S. boulardii* supplementing with STT could improve the eradication rate of *H. pylori*, and concurrently decrease the incidence of total adverse events and gastrointestinal adverse events in children. In the future, it is essential to carry out more large-scale, high-quality, and multicenter RCTs to further investigate the effect of different doses and durations of *S. boulardii* on *H. pylori* eradication in children.

### Electronic supplementary material

Below is the link to the electronic supplementary material.

Supplementary Material 1

## Data Availability

The data presented in the study are included in the original article/Supplementary Material. Further inquiries can be directed to the corresponding author.
