# Supplementary material for: The effect of Saccharomyces boulardii supplementation on Helicobacter pylori eradication in children: a systematic review and meta-analysis of Randomized controlled trials
Source: BMC Infect Dis. 2023 Dec 15;23:878. doi: 10.1186/s12879-023-08896-4 (PMC10722661; doi:10.1186/s12879-023-08896-4)

The Effect of Saccharomyces boulardii Supplementation on *Helicobacter pylori* Eradication in Children: A Systematic Review and Meta-Analysis of Randomized Controlled Trials

Supplementary material

Content		Page
**Table S1.**	Search strings for PubMed	2
**Table S2.**	Description of excluded studies	2-8
**Table S3.**	GRADE evidence profile for H. pylori eradication rate	9
**Table S4.**	GRADE evidence profile	10-14
**Figure S1**.	Risk of bias graph	15
**Figure S2**.	Risk of bias summary	16
**Figure S3**.	Forest plot for specific adverse effects (vomiting, constipation, abdominal pain, epigastric discomfort, and abdominal distention)	17
**Figure S4**.	Forest plot for specific adverse effects (poor appetite, taste disorder, stomatitis, rash)	18
**Figure S5**.	Funnel plot for overall *H. pylori* eradication rate (ITT data)	19
**Figure S6**.	Funnel plot for diarrhea	19
**Figure S7**.	Funnel plot for nausea	20

Table S1 The details of search strategy (from database inception to September 10, 2023)	
**Databases**	**Search strings**
**PubMed**	("helicobacter"[MeSH Terms] OR "helicobacter"[tiab] OR "helicobacter pylori"[MeSH Terms] OR "helicobacter pylori"[tiab] OR "H.pylori"[tiab] OR "Hp"[tiab] OR "campylobacter pylori"[tiab]) AND ("saccharomyces boulardii"[tiab] OR "S. boulardii"[tiab] OR "bioflor"[tiab] OR probiotics[MeSH] OR probiotics[tiab] OR probiotic[tiab]) AND ("children"[tiab] OR "childhood"[tiab] OR "pediatric"[tiab] OR "pediatrics"[tiab] OR "adolescents"[tiab])

Table S2. Description of excluded studies at the stage of eligibility according to the PRISMA flow chart.

No.	First author	Publication year	Reason for exclusion
1.	Li	2009	Non-S. boulardii (Bifidobacterium triple live bacteria)
2.	Szajewska	2010	Meta-analysis
3.	Pan	2010	Non-S. boulardii (lactobacillus)
4.	Zhang	2010	Non-S. boulardii (Bacillus subtilis and Enterococcus faecim, Bifidobacterium tetravalent live bacteria)
5.	Ahmad	2013	Non-S. boulardii (containing lactic acid bacteria and bifidobacteria)
6.	Yang	2013	Non-S. boulardii (Bifidobacterium triple live bacteria)
7.	Zhao	2013	Unreported primary outcome of interest
8.	Li	2014	Meta-analysis
9.	He	2014	Unable to obtain full text
10	Li	2014	Non-S. boulardii (lactobacillus)
11	Szajewska	2015	Meta-analysis
12	Zhong	2015	Non-S. boulardii (Bifidobacterium triple live bacteria)

13	Namkin	2016	Intervention did not meet criteria
14	Fang	2016	Non-S. boulardii (containing lactic acid bacteria and bifidobacteria)
15	Meng	2016	Non-S. boulardii (lactobacillus)
16	Peng	2016	Probiotics unknown
17	Feng	2017	Meta-analysis
18	Huang	2017	Incorrect follow-up time
19	Zou	2017	Non-S. boulardii (Bifidobacterium triple live bacteria)
20	Li	2018	Non-S. boulardii (Bifidobacterium triple live bacteria)
21	Lin	2018	Non-S. boulardii (Bifidobacterium triple live bacteria)
22	Zhang	2018	Non-S. boulardii (Bifidobacterium triple live bacteria)
23	Dong	2019	Incorrect follow-up time
24	Wang	2019	Non-S. boulardii (Compound Lactobacillus acidophilus)
25	Zhang	2019	Non-S. boulardii (Clostridium butyricum binary viable bacteria)
26	Huang	2020	Non-S. boulardii (Bifidobacterium triple live bacteria)
27	Liu	2020	Non-S. boulardii (Bifidobacterium triple live bacteria)
28	Mei	2020	Meta-analysis
29	Qiao	2020	Non-S. boulardii (containing lactic acid bacteria and bifidobacteria)
30	Rong	2020	Non-S. boulardii (Clostridium butyricum live bacteria)
31	Sun	2020	Non-S. boulardii (Clostridium butyricum binary viable bacteria)
32	Wang	2020	Non-S. boulardii (Bifidobacterium triple live bacteria)
33	Wei	2020	Non-RCT
34	Zhang	2020	Non-S. boulardii (Clostridium butyricum and Bacillus coagulans viable bacteria)
35	Zhang	2020	Unable to obtain full text
36	Liu	2020	Incorrect follow-up time
37	Yang	2020	Incorrect follow-up time
38	Karhan	2021	Non-RCT

39	Kang	2021	Non-RCT
40	Li	2021	Non-RCT, Non-S. boulardii
41	Li	2021	Non-S. boulardii (Bifidobacterium triple live bacteria)
42	Li	2021	Incorrect follow-up time
43	Liu	2021	Incorrect follow-up time
44	Liu	2021	Non-S. boulardii (Clostridium butyricum binary viable bacteria)
45	Yang	2021	Non-S. boulardii (Clostridium butyricum binary viable bacteria)
46	Zhao	2021	Unable to obtain full text
47	Cheng	2022	Non-S. boulardii (Bifidobacterium triple live bacteria)
48	Zhai	2022	Non-S. boulardii (Clostridium butyricum binary viable bacteria)
49	Li	2022	Non-S. boulardii (Bifidobacterium triple live bacteria)
50	Liao	2022	Non-S. boulardii (Clostridium butyricum Enterococcus triple live bacteria)
51	Liu	2022	Probiotics unknown
52	Zhao	2022	Unable to obtain full text
53	Zhou	2022	Non-S. boulardii (Bifidobacterium triple live bacteria)

References for the table

1. Li Z. The efficacy of triple therapy and probiotic sequential treatment for Helicobacter pylori infection in children. Chinese Journal of Preventive Medicine, 2009,10 (08): 728-729.

2. Szajewska H, Horvath A, Piwowarczyk A. Meta-analysis: The effects of Saccharomyces boulardii supplementation on Helicobacter pylori eradication rates and side effects during treatment. Alimentary Pharmacology and Therapeutics 2010, 32(9):1069-1079.

3. Pan TT, Zhu H, Lu HJ. Observation on the efficacy of probiotic assisted triple therapy in the treatment of Helicobacter pylori infection in children. Straits Pharmacy, 2010,22 (02): 109-110.

4. Zhang L, Dou HJ, Zhao ZY. Observation of the efficacy of probiotics in the adjuvant treatment of Helicobacter pylori infection in children. Chinese Journal of Modern Medicine, 2010,12 (11): 92-93

5. Ahmad K, Fatemeh F, Mehri N, Maryam S. Probiotics for the treatment of pediatric helicobacter pylori infection: a randomized double blind clinical trial. Iran J Pediatr. 2013;23(1):79-84.

6. Yang Y, Huang JT, Shao SY. Analysis of the efficacy of probiotics combined with triple therapy in the treatment of Helicobacter pylori infection in children. Chinese Journal of Hospital Infection, 2013,23 (23): 5757-5758+5761.

7. Zhao XM, Song QL. Clinical observation of Saccharomyces boulardii in the treatment of Helicobacter pylori

in children. Medical Information, 2013, 26 (6): 397.

8. Li S, Huang XL, Sui JZ, et al. Meta-analysis of randomized controlled trials on the efficacy of probiotics in Helicobacter pylori eradication therapy in children. Eur J Pediatr. 2014;173(2):153-161.

9. He AZ. Observation on the efficacy of probiotics combined with triple therapy in the treatment of Helicobacter pylori infection in children. Chinese and Foreign Health Digest, 2014, 19:122.

10. Li M, Zhou XM. Observation on the efficacy of triple therapy combined with probiotics in the treatment of Helicobacter pylori infection in children. Journal of Modern Integrated Traditional Chinese and Western Medicine, 2014, 23 (26): 2883-2884.

11. Szajewska H, Horvath A, Kołodziej M. Systematic review with meta-analysis: Saccharomyces boulardii supplementation and eradication of Helicobacter pylori infection. Aliment Pharmacol Ther. 2015;41(12):1237-1245.

12. Zhong HZ, Ye YZ, Lv B. Clinical observation of probiotics combined with triple therapy in the treatment of Helicobacter pylori infection in children. Chinese Medical Engineering, 2015,23 (11): 144+146.

13. Namkin K, Zardast M, Basirinejad F. Saccharomyces Boulardii in Helicobacter Pylori Eradication in Children: A Randomized Trial From Iran. Iran J Pediatr. 2016;26(1):e3768.

14. Fan J, Li MN, Liao J. Observation of probiotics combined with triple therapy in the treatment of Helicobacter pylori infection in children. Modern Clinical Medicine, 2016,42 (2): 114-116.

15. Meng XL. Analysis of the efficacy of probiotics and triple therapy in the treatment of children with Helicobacter pylori associated chronic gastritis. Chinese Journal of Health and Nutrition, 2016, 26 (33): 28-29.

16. Peng H, Deng M. Clinical study of probiotics combined with triple therapy in the treatment of Helicobacter pylori associated chronic gastritis in children. Clinical Medical Practice, 2016, 25(10):795-797.

17. Feng JR, Wang F, Qiu X, McFarland LV, Chen PF, Zhou R, Liu J, Zhao Q, Li J. Efficacy and safety of probiotic-supplemented triple therapy for eradication of Helicobacter pylori in children: a systematic review and network meta-analysis. European Journal of Clinical Pharmacology 2017, 73(10):1199-1208.

18. Huang L, Zhang YH. Application and efficacy analysis of the combination therapy of Streptomyces boulardii and triple therapy in the treatment of Helicobacter pylori infection in children. J Chinese Journal of Control of Endemic Diseases 2017, 32 (5): 578.

19. Zou FJ, Su CA. Clinical analysis of triple therapy combined with probiotics in the treatment of pediatric Hp related gastritis. J China Maternal and Child Health Study. 2017, 28 (7): 815-816+835。

20. Li J. Evaluation of the efficacy and safety of triple therapy combined with probiotics in the treatment of pediatric Hp associated gastritis.Shezhi, 2018, 30 (04): 632-633.

21. Lin L, Huang YS. Clinical efficacy of probiotics combined with triple therapy in the treatment of Helicobacter pylori associated chronic gastritis in children. Journal of Clinical Rational Drug Use. 2018, 11 (33): 64-65.

22. Zhang YX.The impact of probiotics combined with triple therapy on clinical efficacy, Hp eradication rate, and recurrence in the treatment of Helicobacter pylori infection in children. J Medical Innovation of China 2018,

15 (2): 29-33.

23. Dong SX. A clinical study on the treatment of Helicobacter pylori infection in children using triple therapy combined with Saccharomyces boulardii. Medical Journal. 2019 (17): 0033.

24. Wang XH, Yang ZR, Ji XJ. Probiotics combined with triple therapy on the eradication rate of Helicobacter pylori infection in children and its impact on their nutrition and development. World Latest Medical Information Digest, 2019,19 (86): 148+153.

25. Zhang XP, Zhu MZ, Jiang YZ. Observation of the efficacy of triple therapy combined with probiotics in the treatment of pediatric chronic gastritis complicated with Helicobacter pylori infection. Modern Digestion and Interventional Diagnosis and Treatment, 2019,24 (07): 777-780.

26. Huang WW, Guo SW. Clinical value of probiotics combined with triple therapy in the treatment of functional dyspepsia caused by Helicobacter pylori infection in children. Jilin Medical Journal, 2020,41 (12): 2946-2948.

27. Liu Y. Analysis of the effect of probiotics on the eradication rate of Helicobacter pylori infection in children and on their nutrition and development. Family Medicine: Medical Selection 2020,7, 387-388.

28. Mei ZJ, Li DD, Luo ZC, et al. The efficacy of triple therapy supplemented with Saccharomyces boulardii in eradicating Helicobacter pylori in children: a meta-analysis of randomized controlled trials. Chinese Journal of Microbiology, 2020,32 (02): 161-165.

29. Qiao SY. Clinical value analysis of triple therapy combined with probiotics in the treatment of pediatric Hp associated gastritis. Journal of Practical Gynecology Endocrinology Electronics, 2020,7 (30): 164+169.

30. Rong JG, Xiao X. Clinical study of probiotic assisted triple therapy in the treatment of pediatric chronic gastritis complicated with Helicobacter pylori infection. Grassroots Medical Forum, 2020,24 (14): 1959-1960.

31. Sun HX, Liu SQ, Liu Y. The efficacy and safety of probiotics in treating chronic gastritis caused by Helicobacter pylori infection in children. Shenzhen Journal of Integrated Traditional Chinese and Western Medicine, 2020,30 (14): 123-124.

32. Wang F, Hu FQ, Wang L, et al. The efficacy of probiotics combined with triple therapy in the treatment of Helicobacter pylori associated gastritis in children and its impact on inflammatory factors. Modern Biomedical Progress, 2020,20 (19): 3797-3800.

33. Wei Li. The efficacy of Bladder's yeast powder in the treatment of late stage HP infection in children. J KANGYI 2020, 4: 211.

34. Zhang J. Clinical research on a new plan of berberine hydrochloride and probiotic eradication of Helicobacter pylori. Master's degree Air Force Military Medical University.2020.

35. Zhang QS. To explore the effect of probiotics combined with triple therapy on the eradication rate of Helicobacter pylori (Hp) infection in children. Dongfang Tonic Diet, 2020, (19): 137.

36. Liu YM. Clinical efficacy observation of triple therapy combined with Bladder's yeast powder in the treatment of Helicobacter pylori infection in children. Journal of Clinical Rational Drug Use, 2020,13 (14): 55-56.

37. Yang Y, Tian YY, Hong ST. The effect of probiotics on the eradication rate of Helicobacter pylori infection in children and on their nutrition and development. J China Foreign Medical Treatment 2020, 39 (10): 105-107.

38. Karhan AN, Kayacan H, Gülseren A, Derici D, Usta Y. Assessment of the efficacy of Saccharomyches boulardii in the treatment of Helicobacter pylori in a single center experience spanning over 10 years. Journal of Pediatric Gastroenterology and Nutrition. 2021, 72(SUPPL 1):755.

39. Kang L. Observation of 148 cases of Helicobacter pylori associated gastric ulcers treated with microecological adjuvant therapy. Chinese Journal of Medicine and Clinical Sciences. 2021, 21 (15): 2637-2638.

40. Li D, Zhang B, Qin S, et al. Analysis of the efficacy of probiotics combined with anti Helicobacter pylori triple therapy in the treatment of Helicobacter pylori positive abdominal allergic purpura in children. Chinese Journal of Modern Medicine, 2021,31 (19): 44-48.

41. Li XD. Clinical efficacy observation of probiotic assisted triple standard regimen in the treatment of Helicobacter pylori infection in children. Journal of Huaihai Medicine. 2021, 39 (6): 636-639.

42. Li WB, Yuan RF, Ma D. The effect of standard triple therapy based on proton pump inhibitors combined with Saccharomyces boulardii on Helicobacter pylori eradication rate and serum cytokines in children with Helicobacter pylori infection. J Chinese Remedies&Clinics 2021, 21 (24): 4017-4019.

43. Liu H, Wu W. Observation on the efficacy of triple therapy combined with yeast in the treatment of pediatric peptic ulcer. Shenzhen Journal of Integrated Traditional Chinese and Western Medicine 2021, 31 (21): 116-118.

44. Liu XH. Observation on the efficacy of triple therapy combined with probiotics in the treatment of pediatric chronic gastritis complicated with Helicobacter pylori infection. Yunnan Medical Journal. 2021, 42 (03): 236-237.

45. Yang M. Observation on the efficacy of triple therapy combined with probiotics in the treatment of pediatric chronic gastritis complicated with Helicobacter pylori infection. Contemporary Medical Journal, 2021,19 (2): 103-104.

46. Zhao H, Li C, Zhang YD, Zhang SS, et al. Analysis on the efficacy and safety of the combination of standard triple therapy with S. boulardii to eradicate Helicobacter pylori infection in children. Oriental Tonic Diet, 2021, (20): 270-271.

47. Cheng RR, Cai X, Wu R. The effect of probiotic adjuvant therapy on serum inflammatory factors and gastrointestinal hormones in children with Helicobacter pylori infection gastritis. J Journal of Practical Hospital Clinical Practice. 2022, 19 (04): 156-159.

48. Zhai RQ, Guo Yq, Yu J, et al. Observation on the efficacy of probiotic assisted triple therapy in the treatment of chronic gastritis combined with Hp infection in children. J Chinese Journal of Practical Medicine 2022, 49 (02): 97-100.

49. Li W, Zhang FX, Gong YH. Probiotics combined with triple therapy for the treatment of Helicobacter pylori associated gastritis in children: efficacy, TNF- α And the value of hs CRP levels. Jilin Medical Journal. 2022, 43 (8): 2128-2130.

50. Liao Q, Tang ZH. Clinical efficacy and inflammatory factor levels of probiotics combined with triple therapy in the treatment of Hp positive chronic gastritis in children. Journal of Mathematical Medicine. 2022, 35(4):

563-566.

51. Liu JY, Li WH, Jia L, et al. Clinical efficacy of triple therapy combined with probiotics on children with Helicobacter pylori infection and its impact on their growth and development. Chinese medicine. 2022, 17 (1): 76-79.

52. Zhao XY. Study on the efficacy and incidence of adverse reactions of probiotics combined with triple therapy in the treatment of Helicobacter pylori associated chronic gastritis in children. Health Must Read, 2022, (26): 208-209.

53. Zhou KH. Analysis of the efficacy and incidence of adverse reactions of probiotics combined with triple therapy in the treatment of Helicobacter pylori associated chronic gastritis in children. Jilin Medicine Journal. 2022, 43 (01): 180-181.

Table S3 GRADE evidence profile for *H. pylori* eradication rate

Quality assessment							No of patients		Effect		Quality	Importance
No of studies	Design	Risk of bias	Inconsistency	Indirectness	Imprecision	Other considerations	*S. boulardii* group	Control	Relative (95% CI)	Absolute		
***H. pylori* eradication rate (ITT)**												
15	randomised trials	serious[1]	no serious inconsistency	no serious indirectness	no serious imprecision	reporting bias[2]	974/1110 (87.7%)	794/1046 (75.9%)	RR 1.14 (1.1 to 1.19)	106 more per 1000 (from 76 more to 144 more)	⊕⊕OO LOW	CRITICAL
								75.8%		106 more per 1000 (from 76 more to 144 more)		

[1] Unclear random sequence generation (8 trials), unclear allocation concealment (15 trials), no or unclear blinding (15 trials).

[2] The results of Egger's test showed that there might be publication bias.

Table S4 GRADE evidence profile for adverse events

Quality assessment							No of patients		Effect		Quality	Importance
No of studies	Design	Risk of bias	Inconsistency	Indirectness	Imprecision	Other considerations	Adverse events	Control	Relative (95% CI)	Absolute		
**Total adverse events**												
6	randomised trials	serious[1]	no serious inconsistency	no serious indirectness	no serious imprecision	none	27/295 (9.2%)	84/288 (29.2%)	RR 0.32 (0.21 to 0.48)	198 fewer per 1000 (from 152 fewer to 230 fewer)	⊕⊕⊕O MODERATE	IMPORTANT
								32.1%		218 fewer per 1000 (from 167 fewer to 254 fewer)		
**Diarrhea**												
13	randomised trials	serious[2]	no serious inconsistency	no serious indirectness	no serious imprecision	reporting bias[3]	156/1060 (14.7%)	324/999 (32.4%)	RR 0.46 (0.37 to 0.56)	175 fewer per 1000 (from 143 fewer to 204 fewer)	⊕⊕OO LOW	IMPORTANT
								31.7%		171 fewer per 1000 (from 139 fewer to 200 fewer)		
**Nausea**												

11	randomised trials	serious[4]	no serious inconsistency	no serious indirectness	no serious imprecision	reporting bias[5]	116/910 (12.7%)	182/854 (21.3%)	RR 0.53 (0.4 to 0.72)	100 fewer per 1000 (from 60 fewer to 128 fewer)	⊕⊕OO LOW	IMPORTANT
								23.3%		110 fewer per 1000 (from 65 fewer to 140 fewer)		
**Vomiting**												
6	randomised trials	serious[6]	serious[7]	no serious indirectness	no serious imprecision	none	142/629 (22.6%)	198/628 (31.5%)	RR 0.67 (0.47 to 0.94)	104 fewer per 1000 (from 19 fewer to 167 fewer)	⊕⊕OO LOW	IMPORTANT
								32.4%		107 fewer per 1000 (from 19 fewer to 172 fewer)		
**Constipation**												
6	randomised trials	serious[8]	no serious inconsistency	no serious indirectness	no serious imprecision	none	49/574 (8.5%)	112/525 (21.3%)	RR 0.42 (0.31 to 0.58)	124 fewer per 1000 (from 90 fewer to 147 fewer)	⊕⊕⊕O MODERATE	IMPORTANT
								12.8%		74 fewer per 1000 (from 54 fewer to 88 fewer)		
**Abdominal pain**												

6	randomised trials	serious[6]	no serious inconsistency	no serious indirectness	no serious imprecision	none	96/684 (14%)	132/635 (20.8%)	RR 0.67 (0.46 to 0.96)	69 fewer per 1000 (from 8 fewer to 112 fewer)	⊕⊕⊕O MODERATE	IMPORTANT
								18.6%		61 fewer per 1000 (from 7 fewer to 100 fewer)		
**Abdominal distension**												
4	randomised trials	serious[9]	no serious inconsistency	no serious indirectness	no serious imprecision	none	21/232 (9.1%)	33/176 (18.8%)	RR 0.47 (0.27 to 0.81)	99 fewer per 1000 (from 36 fewer to 137 fewer)	⊕⊕⊕O MODERATE	IMPORTANT
								18.6%		99 fewer per 1000 (from 35 fewer to 136 fewer)		
**Epigastric discomfort**												
2	randomised trials	serious[10]	no serious inconsistency	no serious indirectness	no serious imprecision	none	7/99 (7.1%)	17/99 (17.2%)	RR 0.41 (0.18 to 0.9)	101 fewer per 1000 (from 17 fewer to 141 fewer)	⊕⊕⊕O MODERATE	IMPORTANT
								20.4%		120 fewer per 1000 (from 20 fewer to 167 fewer)		
**Poor appetite**												

6	randomised trials	serious[1]	no serious inconsistency	no serious indirectness	no serious imprecision	none	52/381 (13.6%)	81/325 (24.9%)	RR 0.5 (0.35 to 0.72)	125 fewer per 1000 (from 70 fewer to 162 fewer)	⊕⊕⊕O MODERATE	IMPORTANT
								25.9%		130 fewer per 1000 (from 73 fewer to 168 fewer)		
**Taste disorder**												
1	randomised trials	serious[11]	no serious inconsistency	no serious indirectness	serious[12]	none	4/41 (9.8%)	8/41 (19.5%)	RR 0.5 (0.16 to 1.53)	98 fewer per 1000 (from 164 fewer to 103 more)	⊕⊕OO LOW	IMPORTANT
								19.5%		97 fewer per 1000 (from 164 fewer to 103 more)		
**Stomatitis**												
2	randomised trials	serious[13]	no serious inconsistency	no serious indirectness	no serious imprecision	none	14/370 (3.8%)	53/370 (14.3%)	RR 0.26 (0.15 to 0.47)	106 fewer per 1000 (from 76 fewer to 122 fewer)	⊕⊕⊕O MODERATE	IMPORTANT
								14.3%		106 fewer per 1000 (from 76 fewer to 122 fewer)		
**Rash**												

4	randomised trials	serious[14]	no serious inconsistency	no serious indirectness	serious[15]	none	2/195 (1%)	8/187 (4.3%)	RR 0.32 (0.09 to 1.18)	29 fewer per 1000 (from 39 fewer to 8 more)	⊕⊕OO LOW	IMPORTANT
								3.8%		26 fewer per 1000 (from 35 fewer to 7 more)		

[1] Unclear random sequence generation (3 trials), unclear allocation concealment (6 trials), no or unclear blinding (6 trials).

[2] Unclear random sequence generation (7 trials), unclear allocation concealment (13 trials), no or unclear blinding (13 trials).

[3] The Begg's test and Egger's test indicate the possibility of publication bias

[4] Unclear random sequence generation (5 trials), unclear allocation concealment (11 trials), no or unclear blinding (11 trials)

[5] The Egger's test suggested that there may be a publication bias.

[6] Unclear random sequence generation (1 trials), unclear allocation concealment (6 trials), no or unclear blinding (6 trials).

[7] $I^2 > 50\%$.

[8]Unclear random sequence generation (2 trials), unclear allocation concealment (6 trials), no or unclear blinding (6 trials).

[9] Unclear random sequence generation (2 trials), unclear allocation concealment (4 trials), no or unclear blinding (4 trials).

[10] Unclear random sequence generation (2 trials), unclear allocation concealment (2 trials), no or unclear blinding (2 trials).

[11] Unclear random sequence generation (1 trial), unclear allocation concealment (1 trial), unclear blinding (1 trial).

[12] Small sample size, wide confidence interval,without excluding appreciable harm or benefit.

[13] Unclear allocation concealment (2 trials), unclear blinding (2 trials).

[14] Unclear random sequence generation (3 trials), unclear allocation concealment (4 trials), unclear blinding (4 trials).

[15] Wide confidence interval

Figure S1. Risk of bias graph: review authors' judgements about each risk of bias item presented as percentages across all included studies.

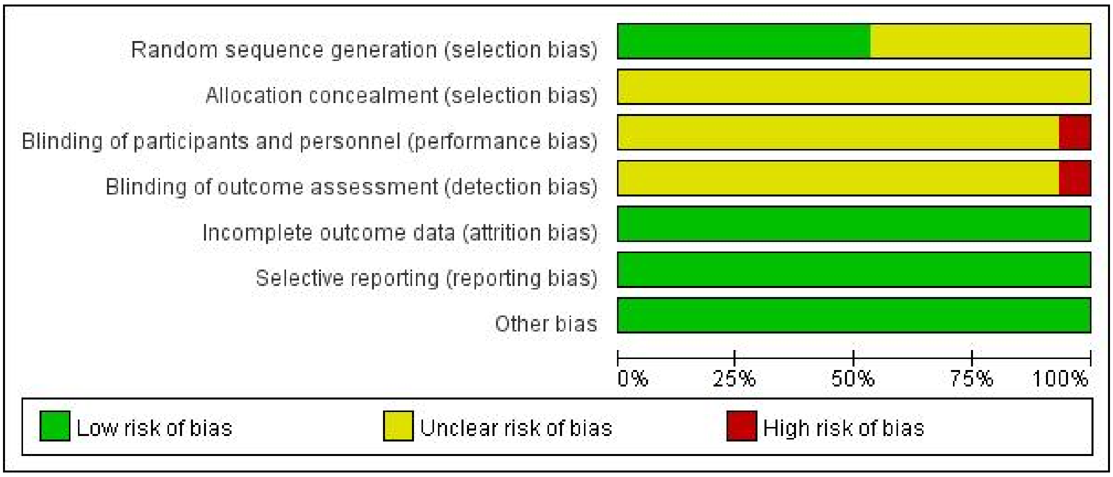

Figure S2. Risk of bias summary: review authors' judgements about each risk of bias item for each included study.

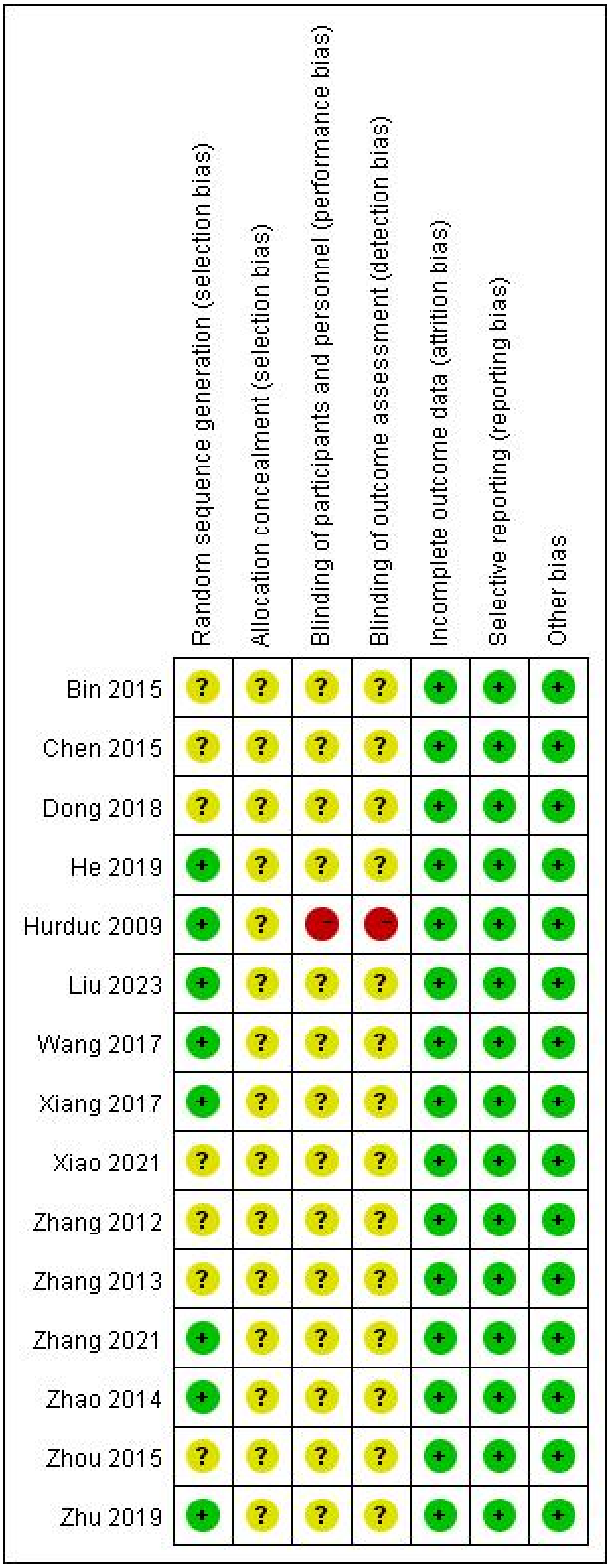

Figure S3. Forest plot for specific adverse effects (vomiting, constipation, abdominal pain, epigastric discomfort, and abdominal distention)

Study or Subgroup	S. boulardii Events	S. boulardii Total	Control Events	Control Total	Weight	Risk Ratio M-H, Random, 95% CI
**2.4.1 Vomiting**						
Chen 2015	12	120	36	120	16.8%	0.33 [0.18, 0.61]
He 2019	10	60	21	60	15.2%	0.48 [0.25, 0.92]
Liu 2023	8	39	6	38	9.5%	1.30 [0.50, 3.39]
Xiang 2017	75	250	87	250	28.5%	0.86 [0.67, 1.11]
Zhao 2014	34	120	42	120	24.2%	0.81 [0.56, 1.18]
Zhu 2019	3	40	6	40	5.8%	0.50 [0.13, 1.86]
**Subtotal (95% CI)**		**629**		**628**	**100.0%**	**0.67 [0.47, 0.94]**
Total events	142		198			
Heterogeneity: Tau² = 0.09; Chi² = 11.87, df = 5 (P = 0.04); I² = 58%						
Test for overall effect: Z = 2.28 (P = 0.02)						
**2.4.2 Constipation**						
Liu 2023	1	39	2	38	1.8%	0.49 [0.05, 5.15]
Xiang 2017	27	250	65	250	58.4%	0.42 [0.27, 0.63]
Zhang 2013	0	30	3	30	1.2%	0.14 [0.01, 2.65]
Zhang 2021	7	95	5	47	8.3%	0.69 [0.23, 2.07]
Zhao 2014	13	120	31	120	28.0%	0.42 [0.23, 0.76]
Zhu 2019	1	40	6	40	2.3%	0.17 [0.02, 1.32]
**Subtotal (95% CI)**		**574**		**525**	**100.0%**	**0.42 [0.31, 0.58]**
Total events	49		112			
Heterogeneity: Tau² = 0.00; Chi² = 2.13, df = 5 (P = 0.83); I² = 0%						
Test for overall effect: Z = 5.37 (P < 0.00001)						
**2.4.3 Abdominal pain**						
Chen 2015	15	120	35	120	21.6%	0.43 [0.25, 0.74]
He 2019	8	60	19	60	15.2%	0.42 [0.20, 0.89]
Liu 2023	1	39	4	38	2.7%	0.24 [0.03, 2.08]
Xiang 2017	42	250	47	250	29.2%	0.89 [0.61, 1.30]
Zhang 2021	10	95	5	47	9.8%	0.99 [0.36, 2.73]
Zhao 2014	20	120	22	120	21.5%	0.91 [0.52, 1.58]
**Subtotal (95% CI)**		**684**		**635**	**100.0%**	**0.67 [0.46, 0.96]**
Total events	96		132			
Heterogeneity: Tau² = 0.08; Chi² = 8.73, df = 5 (P = 0.12); I² = 43%						
Test for overall effect: Z = 2.20 (P = 0.03)						
**2.4.4 Abdominal distension**						
Liu 2023	1	39	3	38	5.9%	0.32 [0.04, 2.99]
Wang 2017	3	57	9	50	18.7%	0.29 [0.08, 1.02]
Zhang 2012	4	41	12	41	26.7%	0.33 [0.12, 0.95]
Zhang 2021	13	95	9	47	48.7%	0.71 [0.33, 1.55]
**Subtotal (95% CI)**		**232**		**176**	**100.0%**	**0.47 [0.27, 0.81]**
Total events	21		33			
Heterogeneity: Tau² = 0.00; Chi² = 2.23, df = 3 (P = 0.53); I² = 0%						
Test for overall effect: Z = 2.73 (P = 0.006)						
**2.4.5 Epigastric discomfort**						
Zhang 2012	6	41	16	41	91.6%	0.38 [0.16, 0.86]
Zhou 2015	1	58	1	58	8.4%	1.00 [0.06, 15.61]
**Subtotal (95% CI)**		**99**		**99**	**100.0%**	**0.41 [0.18, 0.90]**
Total events	7		17			
Heterogeneity: Tau² = 0.00; Chi² = 0.45, df = 1 (P = 0.50); I² = 0%						
Test for overall effect: Z = 2.21 (P = 0.03)						

Test for subgroup differences: Chi² = 5.77, df = 4 (P = 0.22), I² = 30.7%

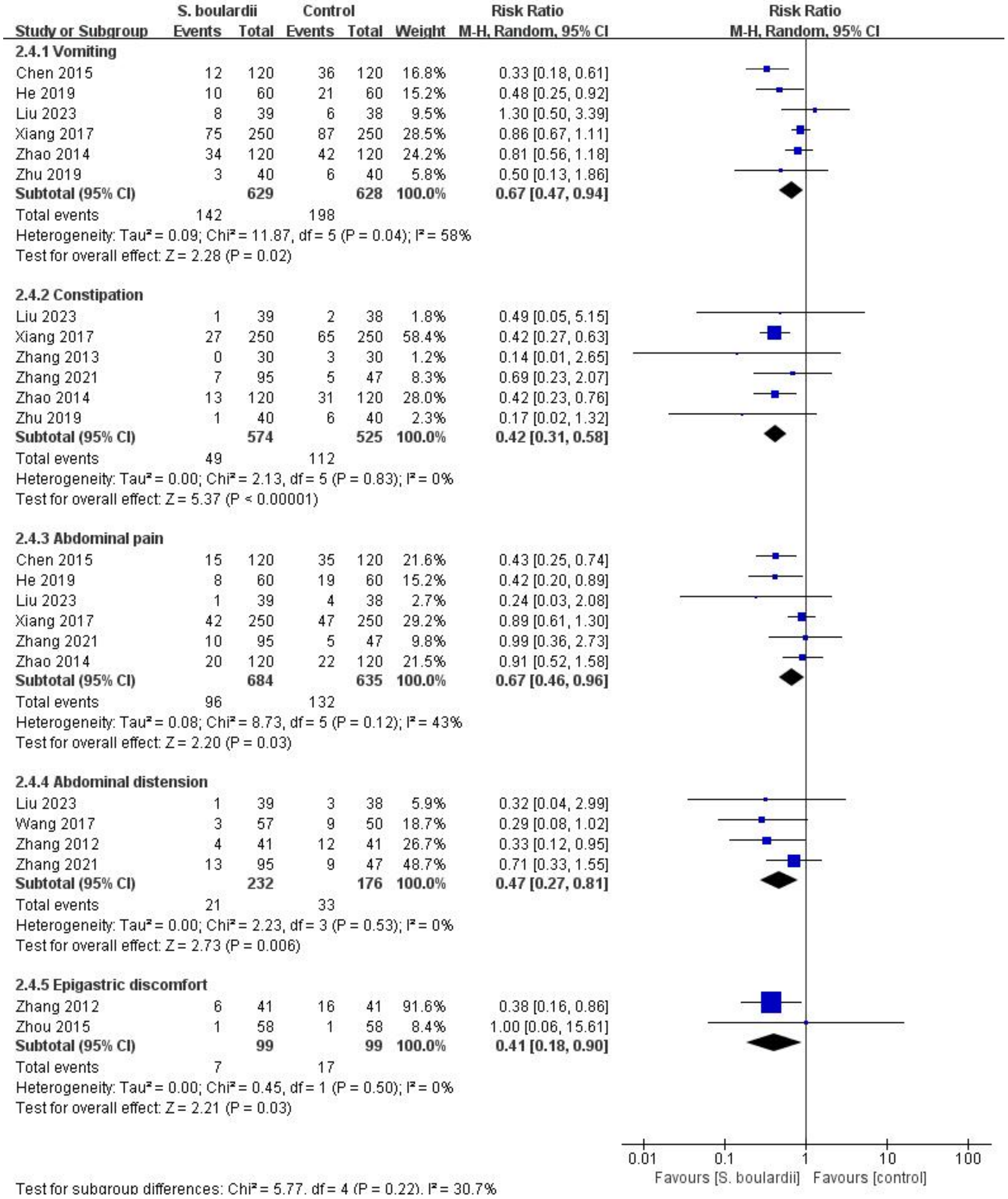

Figure S4. Forest plot for specific adverse effects (poor appetite, taste disorder, stomatitis, rash)

Study or Subgroup	S. boulardii Events	S. boulardii Total	Control Events	Control Total	Weight	Risk Ratio M-H, Random, 95% CI
**2.5.1 Poor appetite**						
Chen 2015	10	120	31	120	24.2%	0.32 [0.17, 0.63]
Liu 2023	1	39	3	38	2.7%	0.32 [0.04, 2.99]
Wang 2017	5	57	13	50	13.1%	0.34 [0.13, 0.88]
Zhang 2013	1	30	2	30	2.4%	0.50 [0.05, 5.22]
Zhang 2021	28	95	18	47	39.5%	0.77 [0.48, 1.24]
Zhu 2019	7	40	14	40	18.1%	0.50 [0.23, 1.11]
**Subtotal (95% CI)**		**381**		**325**	**100.0%**	**0.50 [0.35, 0.72]**
Total events	52		81			
Heterogeneity: Tau² = 0.03; Chi² = 5.77, df = 5 (P = 0.33); I² = 13%						
Test for overall effect: Z = 3.69 (P = 0.0002)						
**2.5.2 Taste disorder**						
Zhang 2012	4	41	8	41	100.0%	0.50 [0.16, 1.53]
**Subtotal (95% CI)**		**41**		**41**	**100.0%**	**0.50 [0.16, 1.53]**
Total events	4		8			
Heterogeneity: Not applicable						
Test for overall effect: Z = 1.21 (P = 0.22)						
**2.5.3 Stomatitis**						
Xiang 2017	10	250	36	250	70.9%	0.28 [0.14, 0.55]
Zhao 2014	4	120	17	120	29.1%	0.24 [0.08, 0.68]
**Subtotal (95% CI)**		**370**		**370**	**100.0%**	**0.26 [0.15, 0.47]**
Total events	14		53			
Heterogeneity: Tau² = 0.00; Chi² = 0.07, df = 1 (P = 0.80); I² = 0%						
Test for overall effect: Z = 4.56 (P < 0.00001)						
**2.5.4 Rash**						
Liu 2023	0	39	1	38	16.8%	0.33 [0.01, 7.74]
Wang 2017	1	57	4	50	36.2%	0.22 [0.03, 1.90]
Zhang 2012	1	41	2	41	30.3%	0.50 [0.05, 5.30]
Zhou 2015	0	58	1	58	16.7%	0.33 [0.01, 8.02]
**Subtotal (95% CI)**		**195**		**187**	**100.0%**	**0.32 [0.09, 1.18]**
Total events	2		8			
Heterogeneity: Tau² = 0.00; Chi² = 0.26, df = 3 (P = 0.97); I² = 0%						
Test for overall effect: Z = 1.71 (P = 0.09)						

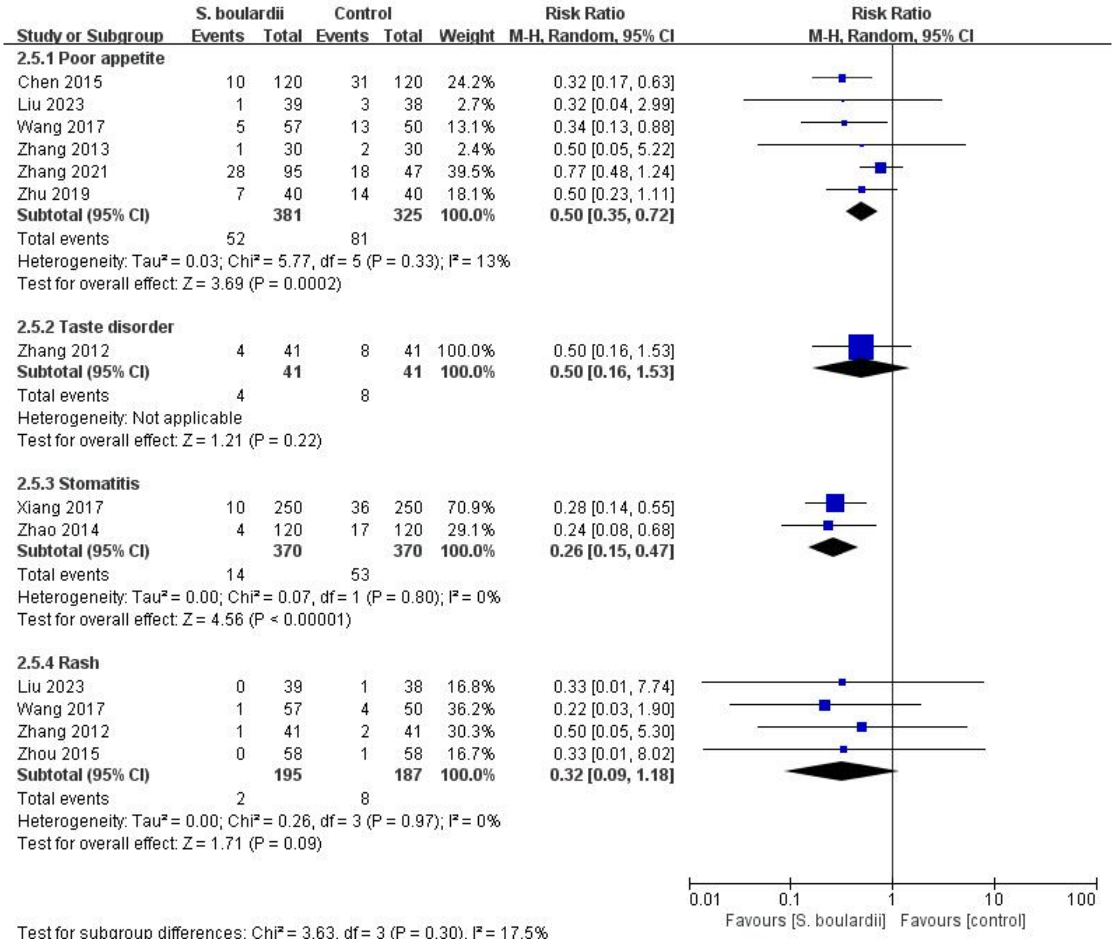

Test for subgroup differences: Chi² = 3.63, df = 3 (P = 0.30), I² = 17.5%

Figure S5. Funnel plot for overall *H. pylori* eradication rate (ITT data)

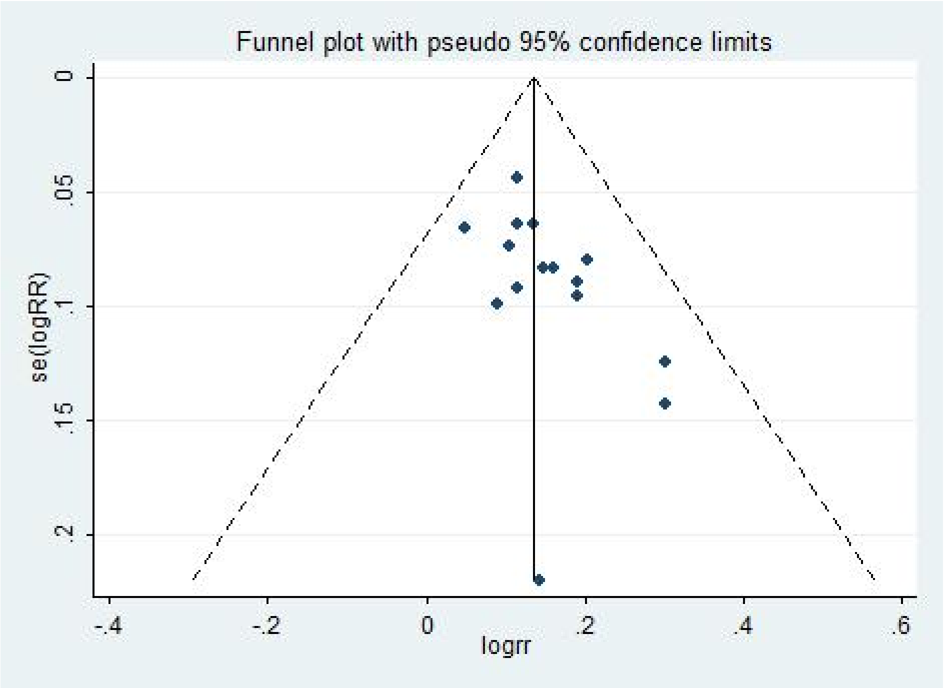

Figure S6. Funnel plot for diarrhea

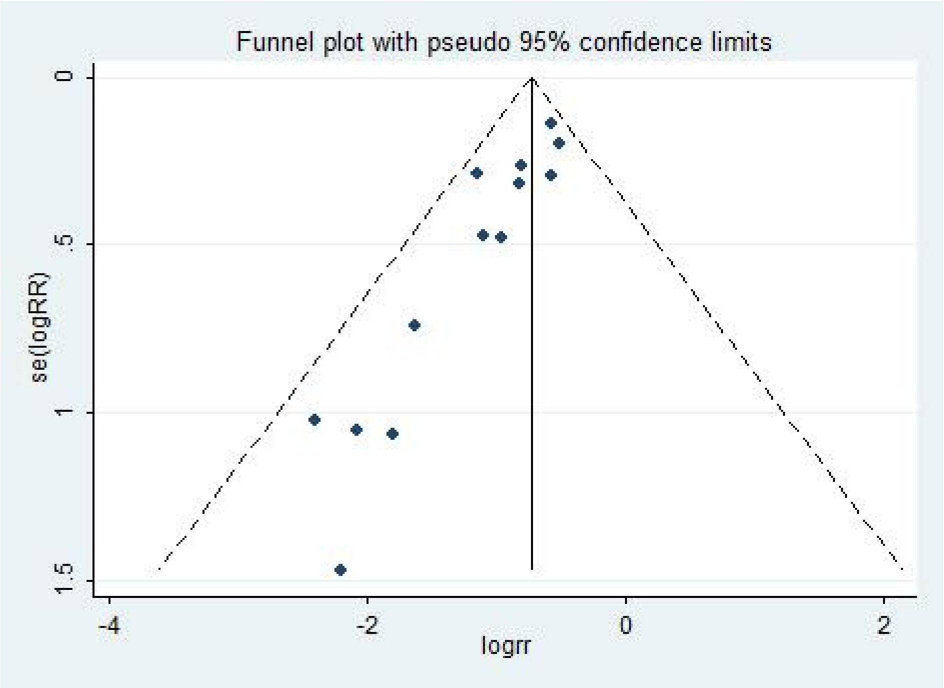

Figure S7. Funnel plot for nausea

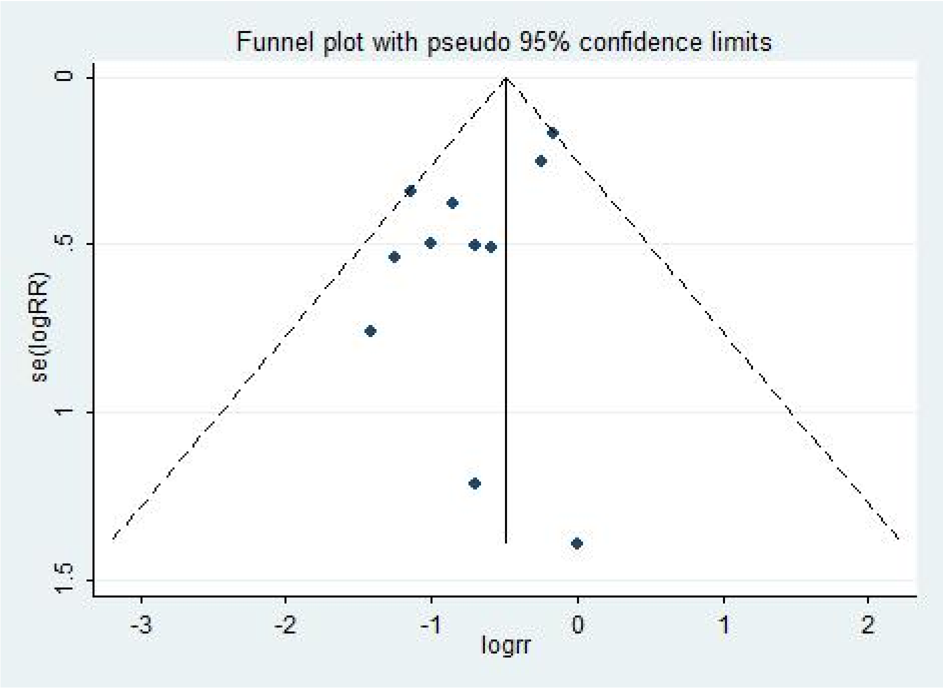

Supplement: Supplementary file 1 — Supplementary Material 1 [file 12879_2023_8896_MOESM1_ESM.pdf]
